# Options for surgical treatment of cervical fractures in patients with spondylotic spine: a case series and review of the literature

**DOI:** 10.1186/s13256-015-0720-7

**Published:** 2015-10-21

**Authors:** Pedro Luz Alves, Delio Eulalio Martins, Renato Hiroshi Salvioni Ueta, David Del Curto, Marcelo Wajchenberg, Eduardo Barros Puertas

**Affiliations:** Department of Orthopedics and Traumatology, Universidade Federal de Sao Paulo, Rua Borges Lagoa, 783, 5th floor, 04038-032 São Paulo, SP Brazil

**Keywords:** Ankylosing, Cervical fractures, Dislocations, Spondylitis, Surgical procedures

## Abstract

**Introduction:**

The surgical treatment of unstable cervical fractures is challenging for spinal surgeons. Unstable cervical fractures associated with spondyloarthropathy and deformities that alter function, form and stability present a greater challenge. Having multiple options to manage this difficult situation is important to all spine surgeons and the results of each case depends on the singular characteristics of patients. The purpose of this case report is to describe the main forms of presentation of unstable cervical subaxial fractures in the spondylotic spine and their surgical treatments.

**Case presentation:**

We present three cases of Caucasian men aged 30, 53 and 59 years with spondylosis with unstable cervical fractures and alternatives choices for surgical treatment, and a review of the literature. Each patient underwent a different surgical procedure of the cervical subaxial spine using an isolated anterior, posterior or combined approach demonstrating good outcomes in all cases.

**Conclusions:**

The treatment of subaxial cervical spinal fractures is complex, and when these fractures are associated with the deformities caused by spondyloarthropathies they can be thought provoking for spine surgeons such as orthopedists or neurosurgeons. The isolated anterior, posterior and combined approaches are safe and effective for treating these pathologies.

## Introduction

The goal of cervical spine surgery is to obtain stability and immediate neural decompression, optimizing the functional outcome and avoiding the complications associated with non-surgical treatment. For cervical fractures associated with spondyloarthropathies and deformities that alter the function, form and stability of the spine, a greater challenge is presented.

Ankylosing spondylitis (AS) is a chronic inflammatory disease that affects the spine. The state of inflammation gradually leads to fusion and ossification of all ligamentous support structures on the vertebrae [1, 2]. Due to osteoporosis and generalized joint stiffness, low-energy trauma can produce severe vertebral fractures, most commonly in the subaxial spine [1, 2].

However, little has been reported regarding the surgical technique for the treatment of fractures of the lower cervical spine in patients with AS [3, 4]. After institutional review board approval (91386/12) and informed consent were signed we conducted a review of the literature and present the main forms of AS presentation and its surgical treatment.

Three cases of cervical fractures in ankylosed columns with different clinical presentations are described.

## Case presentation

### Patient 1

**Fig. 1:**
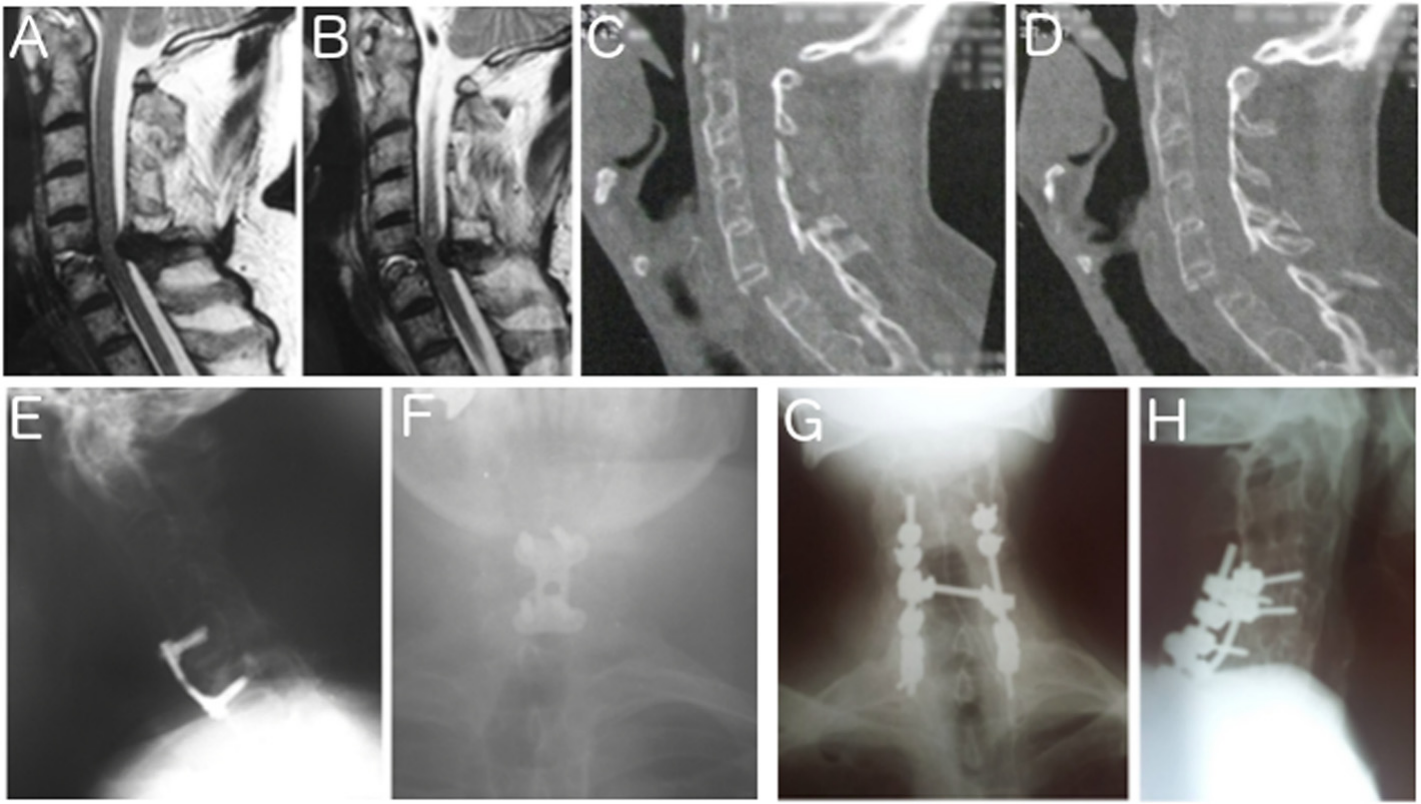
Pre and postoperative images of Patients 1 and 2. Observe in (**a**) and (**b**) the fracture-dislocation of cervical C5 to C6 in the initial sagittal magnetic resonance imaging (T2-weighted) of Patient 1. In (**c**) and (**d**) it is possible to see a fracture-dislocation of C5 to C6 with disruption of anterior and posterior complex in the sagittal computed tomography scan images of Patient 2. **e** Demonstrates the postoperative radiography in lateral view and (**f**) the anteroposterior image demonstrating the short anterior fixation of C5 to C6; note the posterior decompression and stabilization performed in Patient 2 in the anteroposterior (**g**) and lateral (**h**) view of the radiographic images

A 53-year-old Caucasian man presented to our emergency room after a fall in a bathroom complaining of severe neck pain. He had significant joint stiffness in his arms and legs that caused difficulty in walking without assistance. On physical examination, he reported cervical pain and abnormal sensitivity in the left dermatome of C5. Imaging studies showed an ankylosed cervical spine fracture-dislocation at C5 to C6 (Fig. 1).

### Patient 2

A 59-year-old Caucasian man who had a fall presented local pain in his cervical spine with no neurological deficit. Radiographs did not demonstrate fractures. Due to his severe pain and associated AS, a computed tomography (CT) scan was done revealing a fracture-dislocation in C5 to C6 (Fig. 1).

### Patient 3

A 30-year-old Caucasian man with AS developed cervical pain and paraplegia after an automobile collision with a wall. He presented significant pain and deformity in flexion of his cervical region. In addition, he had a tingling sensation in his lower limbs with motor and sensitivity deficits below the C6 level (Fig. 1).

## Discussion

The cervical spine is a complex anatomical structure, and the treatment of lesions in this region is still controversial and challenging for spinal surgeons. Columns afflicted by AS become stiff and more susceptible to fractures, with lesions occurring mostly in the cervical region. Furthermore, there is a high rate of neurological impairment [1–4].

Without established guidelines for the best treatment of these fractures, reports of surgical treatment have increased the extent to which new fixation devices are used during surgery [4].

The surgical methods for fractures of the subaxial cervical spine include the anterior approach with the use of plates, the posterior approach with lateral mass screws and the combined approach. In addition, there are some reports of the use of corrective osteotomies for kyphotic deformities found in patients with spondyloarthropathies [3–5]. Complications specific to AS must be considered when opting for open treatment, such as the increased risk of epidural bleeding and the widespread presence of osteoporotic bone, as well as multi-segmental fusion of long segments of the spine, which is often combined with sagittal deformity and makes the surgery more complex [6]. It is important to emphasize that the surgical approach for each patient discussed here was chosen based on the surgeons’ experience.

In the first clinical case, the patient presented local pain with sensitivity deficit of C5 dermatome. On the initial evaluation, no injuries were identified in the radiographs. Due to local pain, neurological deficit and the associated AS, a magnetic resonance imaging (MRI) scan was done revealing a fracture in the C5 to C6 vertebrae. Some researchers have reported difficulties in visualizing fractures in patients with spondylotic cervical spine, and additional imaging examinations are important to confirm the diagnosis [6, 7].

In this case, because the patient presented a neurological deficit the MRI was done as the primary examination to rule out any intervertebral disc, posterior ligament injury or occult bone fracture [8] allowing surgeons to decide treatment with lower costs.

The isolated anterior approach was chosen because although ligamentum flavum bulging was noted, the most important lesion was in the anterior column. The patient reported a continued improvement of pain without neurological deterioration and an early return to daily activities (Fig. 1).

The anterior decompression technique is associated with less than 2% of significant complications related specifically to the use of the plate, and it provides excellent fusion rates (98.9%) that occur, on average, 3.2 months after surgery [9]. Anterior decompression can promote stability, safety and rigidness, improving neurologic outcomes satisfactorily and representing a good option for early surgery [9, 10].

The anterior plate is predominantly used with anterior lesions but also can be used to treat posterior injuries when performed properly. However, several authors advocate the use of a posterior procedure over an anterior one because they argue that an anterior approach alone should not be performed for predominantly posterior lesions [10].

In our second case we chose posterior decompression and stabilization (Fig. 1) with a good clinical outcome, resulting in an improvement in the patient’s pain and sensibility.

Cervical spinal fractures in patients with AS can be adequately treated with lateral mass plating or interspinous wiring of an autologous rib graft. Adequate postoperative immobilization can be attained with a cervical collar and does not require a halo vest [11, 12].

Nakashima *et al.* [13] demonstrated no neurological deterioration after a posterior open reduction, even in cases of traumatic cervical disc herniation. The favorable clinical and radiological results were obtained through a primary posterior procedure.

In our third clinical case the patient developed cervical pain and paraplegia after an automobile collision with a wall. He presented significant pain, deformity in flexion of the neck and a medullary lesion.

**Fig. 2:**
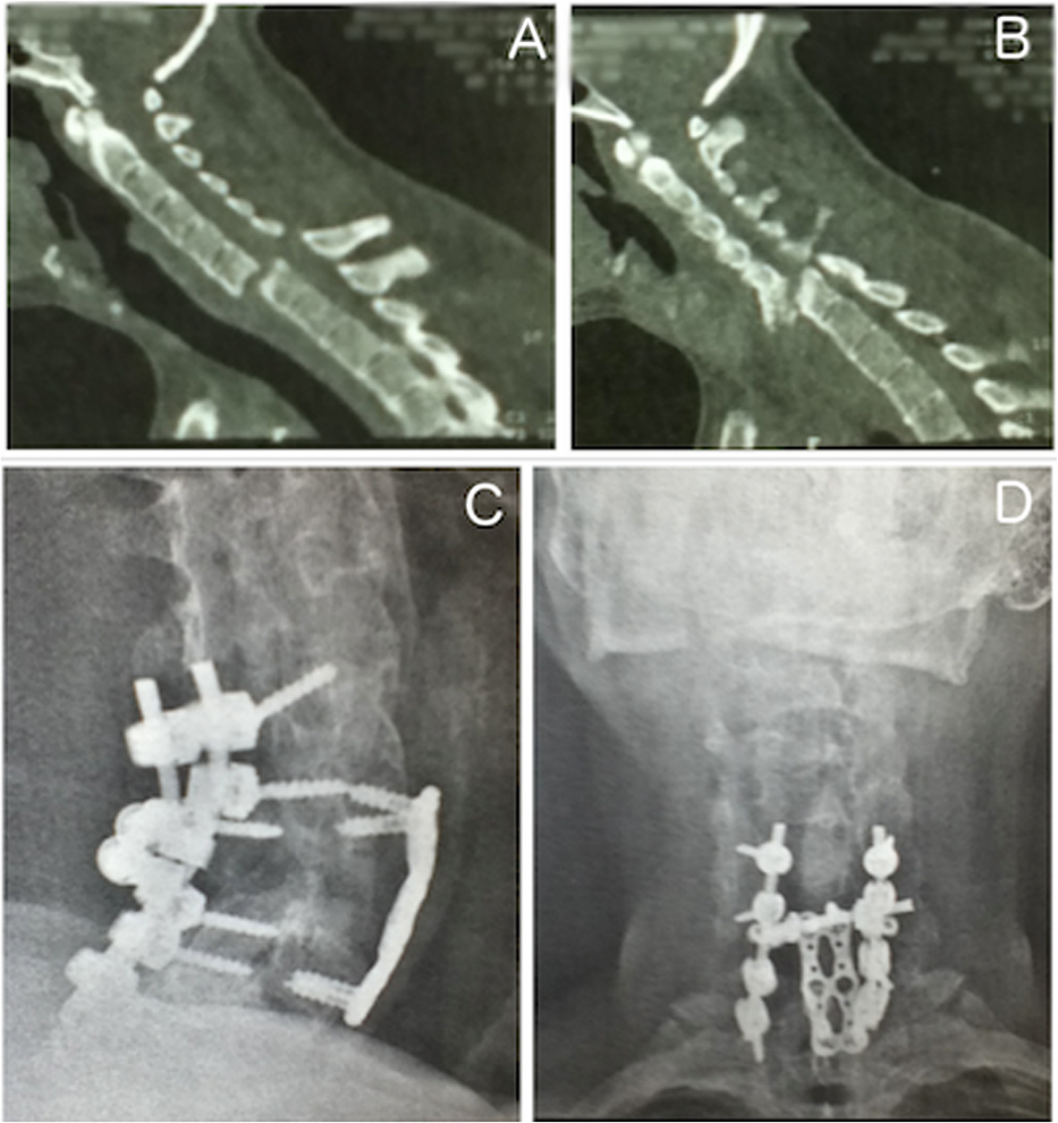
Clinical and surgical presentation of Patient 3. Observe in (**a**) and (**b**) the sagittal computed tomography scan images showing ankylosed cervical spine and a fracture-dislocation of C6 to C7. In (**c**) and (**d**), the radiographic images in sagittal (**c**) and posteroanterior (**d**) views demonstrating the stabilization in 360 degrees

In this case, we chose the dual approach, performing decompression and stabilization using a posterior approach with lateral mass screws associated with the anterior plate, screws and an iliac graft (Fig. 2) in order to guarantee a complete decompression of the medulla and better positioning of his neck.

The patient showed improvement in postoperative pain and in the neurological deficits of his upper limb, predominantly at C6 and C7, but his motor deficit below C8 persisted. Furthermore, he reported improvement in his head position. Before surgery, his head was tilted downward. During the fracture treatment, a deformity correction was performed with placement of an anterior wedge iliac graft, producing a satisfactory jaw–neck angle.

Some researchers have taken the opportunity of cervical trauma to perform a surgical extension osteotomy at the lower cervical spine fracture site by using stretching with a halo for the correction of a flexion deformity of the spine in AS after traumatic injury [5]. However, conservative treatment of fractures in patients with AS can have serious complications, such as infection and loosening pins, brain hemorrhages and pseudoarthrosis, as demonstrated by Schroder *et al.* [14]. We agree with some authors who suggest that the anterior and posterior combined approach is stable and provides immediate decompression. Furthermore, this option is a reasonable surgical strategy for fracture-dislocations of the cervical spine in patients with AS [4, 15].

## Conclusions

The surgical treatment of spondylotic patients with subaxial unstable fractures can be performed successfully by different ways and must be individualized for each patient depending on the clinical evolution and experience of the surgeon. All approaches can be performed in a safe and successful manner just bearing in mind the characteristics of the patients and the lesions.

## Consent

Written informed consent was obtained from the patients for publication of this case report and accompanying images. Copies of the written consents are available for review by the Editor-in-Chief of this journal.
